# Case Report: Reversible myelodysplastic syndrome secondary to sodium valproate in an epileptic child

**DOI:** 10.3389/fped.2026.1828816

**Published:** 2026-06-17

**Authors:** Hongyu Huang, Jiao Chen, Lei Ye, Dan Yu

**Affiliations:** 1 Department of Pediatrics, WCSUH-Tianfu, Sichuan Provincial Children’s Hospital, Meishan, China; 2 Department of Nursing, West China Second University Hospital, Sichuan University, Chengdu, China; 3 Key Laboratory of Birth Defects and Related Diseases of Women and Children, Ministry of Education, Sichuan University, Chengdu, China; 4 Department of Laboratory Medicine, West China Second University Hospital, Sichuan University, Chengdu, China; 5 Department of Medical Genetics, West China Second University Hospital, Sichuan University, Chengdu, China

**Keywords:** case report, children, epilepsy, myelodysplastic syndrome, reversible, sodium valproate

## Abstract

**Background:**

We report a rare case of sodium valproate (VPA)-induced reversible myelodysplastic syndrome (MDS) in an Asian child with epilepsy, thus expanding the recognition of VPA-associated hematological toxicity. Although VPA has been widely used, the reversibility of MDS as a complication remains underreported, particularly in pediatric populations.

**Case summary:**

A 9-year-old boy with epilepsy developed pancytopenia (hemoglobin level: 77 g/L, platelet count: 83 × 10^9^/L) and bone marrow-confirmed MDS after 6 months of VPA therapy. Following a reduction in VPA dose to 250 mg/day with adjunctive lacosamide, hematological parameters normalized within 3 months, and a repeat bone marrow examination revealed resolution of dysplastic features. Seizure control was maintained without relapse. This case highlights the dose-dependent nature of VPA-induced MDS and demonstrates its reversibility upon therapeutic intervention.

**Conclusion:**

The reversibility of VPA-induced MDS following dose adjustment underscores the importance of vigilant hematological monitoring in children.

## Introduction

Epilepsy is one of the most common chronic neurological disorders in children, with an annual incidence ranging from 33.3 to 82 per 100,000 individuals (1). Globally, approximately 10.5 million children under 15 years of age have epilepsy, with over 80% residing in low- and middle-income countries (2). The incidence of epilepsy peaks during infancy and decreases through adolescence. In response to this burden, antiepileptic drugs (AEDs) have long served as the mainstay of treatment for epilepsy (3). Current studies have shown that 27 medications have been approved for the treatment of epilepsy, and nearly two-thirds of pediatric epilepsy cases could be well controlled by using AEDs (1, 3). Sodium valproate (VPA), one of the earliest antiepileptic medications, remains a highly effective treatment for both generalized and focal epilepsy in children and adults (4, 5). Despite its therapeutic value, VPA carries a risk of potentially toxic effects. The most common adverse reactions include weight gain, nausea, headache, dizziness, drowsiness, and ataxia (2, 4, 6). Serious complications—including hemorrhagic pancreatitis, hepatotoxicity, and thrombocytopenia—have also been linked to VPA use. Encephalopathy and elevated gamma-glutamyl transferase levels have been further documented.

In the literature, few reports have described the incidence of myelodysplastic syndrome (MDS) in epileptic children treated with VPA, especially in Asia. Recent real-world studies have further confirmed the widespread use of VPA as initial monotherapy in pediatric epilepsy, with a large cohort of 1,943 children demonstrating its general safety profile while highlighting the need for platelet monitoring (7). Therefore, we report a case of an epileptic child with reversible MDS induced by treatment with VPA.

## Case report

### Case description

A 9-year-old boy presented with two episodes of generalized tonic‒clonic seizures in September 2021, each lasting 1–2 min and manifesting as whole-body rigidity, limb twitching, upward gaze, perioral cyanosis, and unresponsiveness. Electroencephalography (EEG) revealed generalized spike-and-slow wave complexes. Baseline laboratory investigations, including complete blood count and hepatic/renal function tests, were unremarkable. The child had no notable past medical history. His development was normal for his age. There was no family history of blood disorders or bone marrow failure. He lived with his parents, who reported good medication adherence. The patient was diagnosed with epilepsy and started on VPA oral solution (250 mg twice daily). Table 1 summarizes the treatment timeline and key clinical events.

**Table 1:** Treatment timeline and key clinical events.

Timeline	Therapeutic interventions	Key clinical events
September 2021	Valproate oral solution 250 mg twice daily	Two episodes of generalized tonic‒clonic seizures; EEG showed generalized spike-and-wave complexes
March 2022	Maintained original dose	No seizures; normal complete blood count (CBC), liver/kidney function, and blood drug levels
April 2022	Valproate reduced to 250 mg once daily, vitamin B12, folate, and B6 supplementation; bone marrow transplantation recommended (not implemented)	Pancytopenia developed; bone marrow biopsy confirmed myelodysplastic syndrome (MDS)
August 2022	Lacosamide added	CBC normalized; Bone marrow morphology restored; EEG showed abnormal epileptiform discharges
November 2022 follow-up	Continued valproate 250 mg + lacosamide	No seizures; sustained normalization

Six months after treatment, the child remained seizure-free and presented normal findings on repeat EEG, routine hematological investigations, liver and kidney function tests, and VPA levels. In April 2022, the child developed limb weakness and poor complexion without fever, hematemesis, melena, or other symptoms.

### Diagnostic assessment

**Figure 1:**
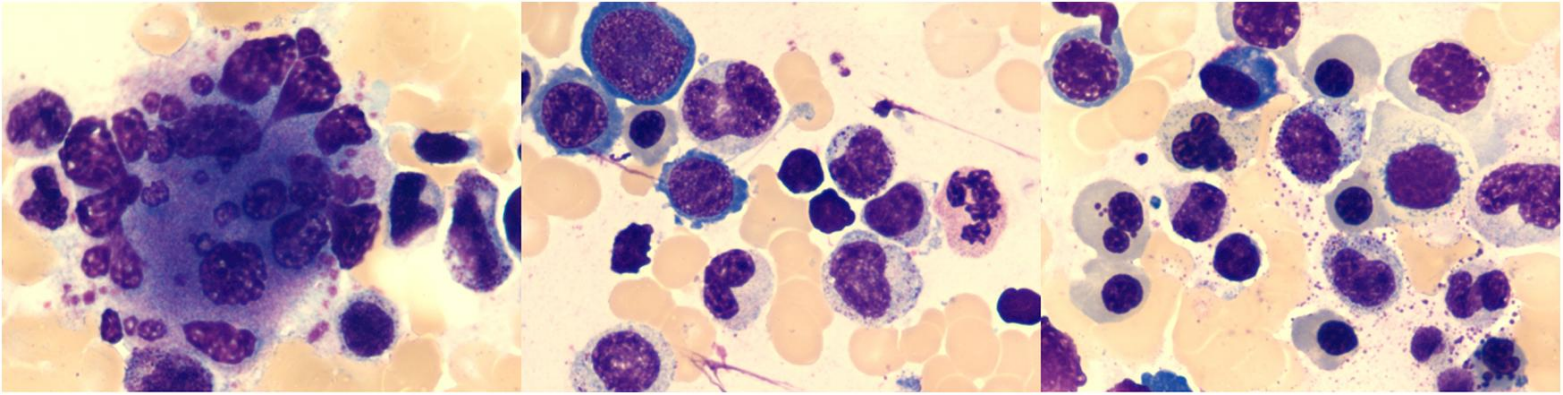
Bone marrow aspirate at diagnosis (April 2022). The smear shows dysplastic features consistent with myelodysplastic syndrome, including multilobular nucleated megakaryocytes, multilobular granulocytes, Howell-Jolly body, etc. (Wright-Giemsa stain, ×400).

Routine blood examination revealed pancytopenia (eosinophils 0.01 × 10^9^/L, monocytes 9.7%, eosinophils 0.01%, erythrocyte count 1.85 × 10^12^/L, hemoglobin 77 g/L, and platelet count 83 × 10^9^/L). A bone marrow examination was performed, and the findings suggested myelodysplastic syndrome (Figure 1). Given the diagnosis, the hematologist recommended oral vitamin B12, folic acid, and vitamin B6, with bone marrow transplantation reserved as an option should medical treatment fail.

### Therapeutic intervention

Because the child's epilepsy had been well controlled with valproate, and given evidence that drug-induced pancytopenia may resolve with dose reduction (8), we reduced the VPA dose from 250 mg twice daily to 250 mg once daily instead of proceeding directly to bone marrow transplantation. We chose this 50% dose reduction for two reasons: first, to maintain some seizure protection while testing for reversibility; second, to minimize the risks of a sudden AED switch. When the lower dose alone later led to EEG abnormalities, we added lacosamide, which has no known hematologic side effects and is effective in children with focal and generalized epilepsies.

**Figure 2:**
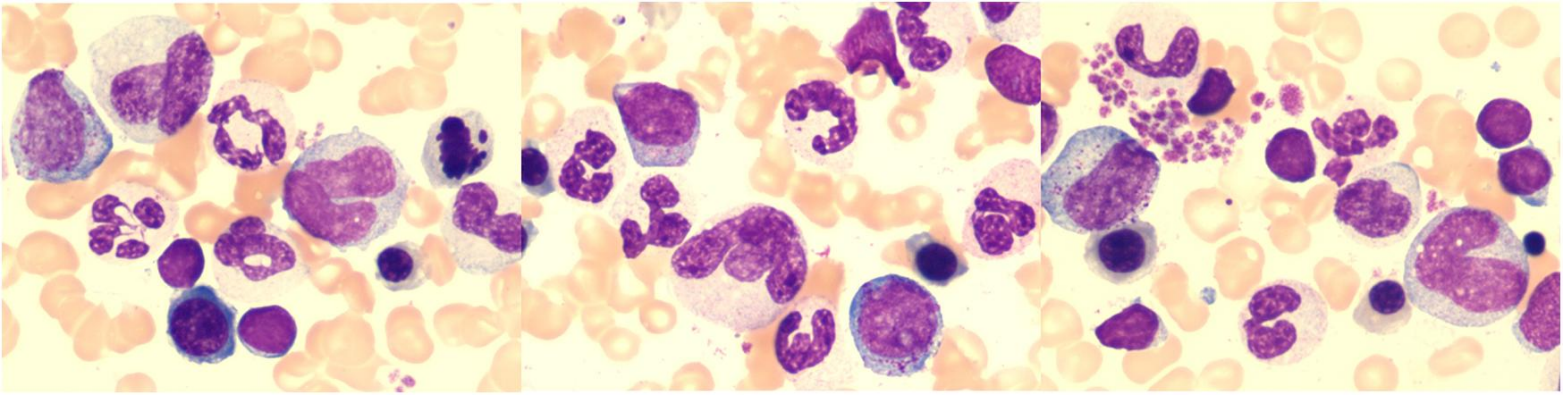
Bone marrow aspirate after VPA dose reduction (August 2022). The smear shows normalized hematopoietic elements with resolution of dysplastic features (Wright-Giemsa stain, ×400).

At 3 months following dose reduction, routine hematological parameters had normalized. A repeat bone marrow examination in August 2022 revealed no obvious abnormalities (Figure 2). Table 2 summarizes the key laboratory values across the three phases. However, EEG re-examination at this time demonstrated obvious epileptic waves.

**Table 2:** Key laboratory values across the three phases.

Parameter	Baseline	MDS phase (April 2022)	Recovery phase (August 2022)	Reference range
Hemoglobin (g/L)	Normal	77	Normal	115–150
Platelet count (×10^9^/L)	Normal	83	Normal	150–400
RBC count (×10^12^/L)	Normal	1.85	Normal	4.0–5.5
Monocyte percentage	Normal	9.7	Normal	3–8
Eosinophil percentage	Normal	0.01	Normal	0.4–8.0
Eosinophil absolute (×10^9^/L)	Normal	0.01	Normal	0.02–0.5
Bone marrow morphology	Not performed	Dysplastic hematopoiesis	Normal	Normal trilineage hematopoiesis without dysplasia
EEG	Abnormal	Normal	Abnormal → improved	Normal brain activity

Normal indicates values within the reference range shown in the final column.

### Follow-up and outcomes

During the 3 months after VPA dose reduction, the child remained clinically stable, with no fever, bleeding, or signs of infection. Monthly hematological monitoring was not performed because the child was school-aged and the family lived far from the hospital. Given the absence of concerning symptoms, we re-assessed bone marrow recovery at 3 months, which confirmed complete resolution of dysplasia.

Subsequently, EEG findings improved, and the child remained seizure-free, while regular hematological parameters remained normal.

## Discussion

MDS resembles acute leukemia in that it involves neoplastic transformation of pluripotent stem cells; it represents a group of clonal disorders of hematopoietic stem cells (9). The pathogenesis of VPA-induced MDS may involve histone deacetylase-mediated epigenetic dysregulation, as evidenced by the synergistic proapoptotic effects of VPA on MDS cells (10). However, MDS induced by sodium valproate in epileptic children has rarely been reported. The occurrence of MDS in our patient might be related to changes in the quantity and quality of blood cells and platelets (8). Notably, our patient's complete hematological recovery following VPA dose reduction supports the hypothesis of dose-dependent toxicity (11). The dose-dependent nature of VPA-induced hematological toxicity is further supported by recent evidence identifying baseline platelet count as an independent risk factor for thrombocytopenia in children receiving VPA therapy (12). The child had normal blood counts and good seizure control while receiving valproate. Dose reduction, however, triggered EEG abnormalities. Therefore, combined treatment with lacosamide and a reduced dose of VPA was initiated, which effectively treated the patient's epilepsy while preventing the undesirable, serious side effects associated with AED therapy. Given the long-term nature of AED therapy, regular monitoring—including blood counts, liver and kidney function, and serum drug levels—is warranted (13).

We cannot completely rule out that the MDS occurred by chance and was unrelated to VPA. However, several findings suggest that VPA was the most likely cause. First, the hematological abnormalities and bone marrow problems developed 6 months after starting VPA, whereas blood counts had been normal before treatment. Second, after we simply reduced the VPA dose (without transplant or chemotherapy), everything returned to normal within 3 months. Third, we checked for and excluded other causes of MDS, such as prior chemotherapy, radiation exposure, other drugs, infections, or inherited bone marrow disorders. Finally, VPA is known to affect bone marrow through histone deacetylase (HDAC) inhibition, which provides a biological explanation. While a chance occurrence is theoretically possible, we believe VPA was the most likely cause in this patient. This case challenges current practice, demonstrating that in selected patients, modification of drug therapy may eliminate the need for invasive procedures like bone marrow transplantation. The treatment for epilepsy in children is usually long-term. Therefore, choosing appropriate AEDs and dosages is key to achieving optimal efficacy with fewer side effects. Our case raises awareness among pediatricians who regularly manage children with epilepsy that pancytopenia may be resolved by adjusting the AED regimen (VPA) and that invasive procedures such as bone marrow may be avoided initially. A larger observational study with a longer follow-up is warranted to validate our observations. This case suggests that hematological complications should prompt re-evaluation of the AED regimen before aggressive procedures are considered.

## Patient perspective

The mother of the patient recalled being “very scared” when she first noticed that her son appeared pale and weak. She described the period surrounding the bone marrow examination as a difficult time but felt relieved when the doctors explained that the condition might improve with a simple dose adjustment rather than a transplant. After we reduced the sodium valproate dose, she watched her son gradually regain his energy, and the blood tests kept coming back normal. She was also glad that his seizures never returned, even with the lower dose. She hopes that by sharing their story, other families facing similar worries might feel less alone.

## Data Availability

The datasets presented in this article are not readily available because of ethical and privacy restrictions. Requests to access the datasets should be directed to the corresponding authors.
